# Effective nose-to-brain delivery of exendin-4 via coadministration with cell-penetrating peptides for improving progressive cognitive dysfunction

**DOI:** 10.1038/s41598-018-36210-9

**Published:** 2018-12-05

**Authors:** Noriyasu Kamei, Nobuyuki Okada, Takamasa Ikeda, Hayoung Choi, Yui Fujiwara, Haruka Okumura, Mariko Takeda-Morishita

**Affiliations:** Laboratory of Drug Delivery Systems, Faculty of Pharmaceutical Sciences, Kobe Gakuin University, 1-1-3 Minatojima, Chuo-ku, Kobe, Hyogo, 650-8586 Japan

## Abstract

In a recent study, we demonstrated the potential of a cell-penetrating peptide (CPP) penetratin to deliver the peptide drug insulin to the brain via nasal administration, and its pharmacological effect on the mild cognitive dysfunction in senescence-accelerated mouse (SAMP8). However, the therapeutic potential of intranasal insulin administration was attenuated when applied to the aged SAMP8 with severe cognitive dysfunction. The present study, therefore, aimed to overcome the difficulty in treating severe cognitive dysfunction using insulin by investigating potential alternatives, glucagon-like peptide-1 (GLP-1) receptor agonists such as exendin-4. Examination using normal ddY mice demonstrated that the distribution of exendin-4 throughout the brain was dramatically increased by intranasal coadministration with the L-form of penetratin. The activation of hippocampal insulin signaling after the simultaneous nose-to-brain delivery of exendin-4 and an adequate level of insulin were confirmed by analyzing the phosphorylation of Akt. Furthermore, spatial learning ability, evaluated in the Morris water maze test after daily administration of exendin-4 with L-penetratin and supplemental insulin for 4 weeks, suggested therapeutic efficacy against severe cognitive dysfunction. The present study suggests that nose-to-brain delivery of exendin-4 with supplemental insulin, mediated by CPP coadministration, shows promise for the treatment of progressive cognitive dysfunction in SAMP8.

## Introduction

The occurrence of dementia, such as during Alzheimer’s disease (AD), has grown with increasing average age in human society. Such diseases and disorders disturb the memory and cognition of patients and are currently seen as a global health concern, which affect the quality of life (QOL) of patients and their families. We currently have very limited options in terms of medicines that symptomatically delay the development of dementia in early stages without curing the disease. Examples include choline esterase inhibitors and N-methyl-D-aspartate (NMDA) receptor antagonists. In the past few decades, while several pharmaceutical companies have taken up the challenge of developing such medicines, effective candidate drugs that could potentially ameliorate memory loss and cognitive dysfunction have still not been found. Among others, antibody drugs and β-secretase (BACE) inhibitors that can reduce amyloid β (Aβ) accumulation in the brain and blood have been tested for AD treatment, as Aβ is considered one of the primary pathogenic molecules in dementia-related disorders. However, such candidate drugs have typically failed to show the expected therapeutic effects in humans, presumably because of their ineffective pharmacological activity and their low efficiency of distribution to the brain parenchyma^1–5^. In particular, current studies suggest that the modulation of Aβ by the action of these drugs might not even contribute to the treatment of AD, because AD is associated with irreversible functional disorder^1,4,6^. Thus, the therapeutic potential of Aβ-lowering medicines such as Aβ antibodies and BACE inhibitors is an ongoing debate.

Recently, AD has been recognized as type 3 diabetes occurring in the brain, and anti-diabetic peptide drugs, such as insulin and glucagon-like peptide-1 (GLP-1) receptor agonists, have been suggested to enhance memory and learning via insulin receptor signaling and resultant glucose uptake into the hippocampal neuronal cells^7–15^. Therefore, such anti-diabetic agents can be considered a new class of potential drugs for dementia. In general, drug transport to the brain is significantly limited by the blood-brain barrier (BBB), consisting of microvascular endothelial cells, pericytes, astrocytes, and others. Meanwhile, some studies further demonstrated that the therapeutic effect of insulin on early-stage dementia could be accomplished by its intranasal administration^8,9^. Nasal administration is currently considered an ideal route for delivering peptide drugs to the central nervous system (CNS), as many researchers have suggested that drugs administered intranasally could be efficiently delivered to the brain by bypassing the BBB^16–19^.

We recently found that a non-covalent strategy with penetratin, one of the typical cell-penetrating peptides (CPPs), could enhance the efficiency of direct nose-to-brain delivery of insulin^20,21^. Moreover, we demonstrated that repeated intranasal administration of insulin with penetratin comprising all L-amino acids (L-penetratin) for 8 weeks could suppress the progress of mild cognitive dysfunction in the senescence-accelerated mouse prone-8 (SAMP8) at 16–24 weeks of age^22^. This suggests that the coadministration of insulin with L-penetratin is a useful way to facilitate nose-to-brain delivery of insulin and further to prevent and cure dementia in the early stage. However, this strategy could not contribute to the recovery of severe cognitive dysfunction during the developed to the progressive stage^22^. Our data suggested that excess delivery of insulin by coadministration with L-penetratin might induce an increase in Aβ accumulation in the brain of SAMP8 with Aβ-related progressive cognitive dysfunction^22^. This unexpected result might be explained by the competition between insulin and Aβ in degradation via insulin-degrading enzyme (IDE) in the brain^23–25^. The accumulated Aβ in a soluble oligomer or an insoluble plaque might reduce the expression level of insulin receptor on the neuronal cell membrane via unfavorable internalization of receptors and induce neuronal degeneration. Therefore, our previous work concluded that efficient delivery of insulin to the brain is an effective therapy in the early stage of dementia, whereas it is unfavorable in the severe stage with Aβ accumulation.

**Figure 1:**
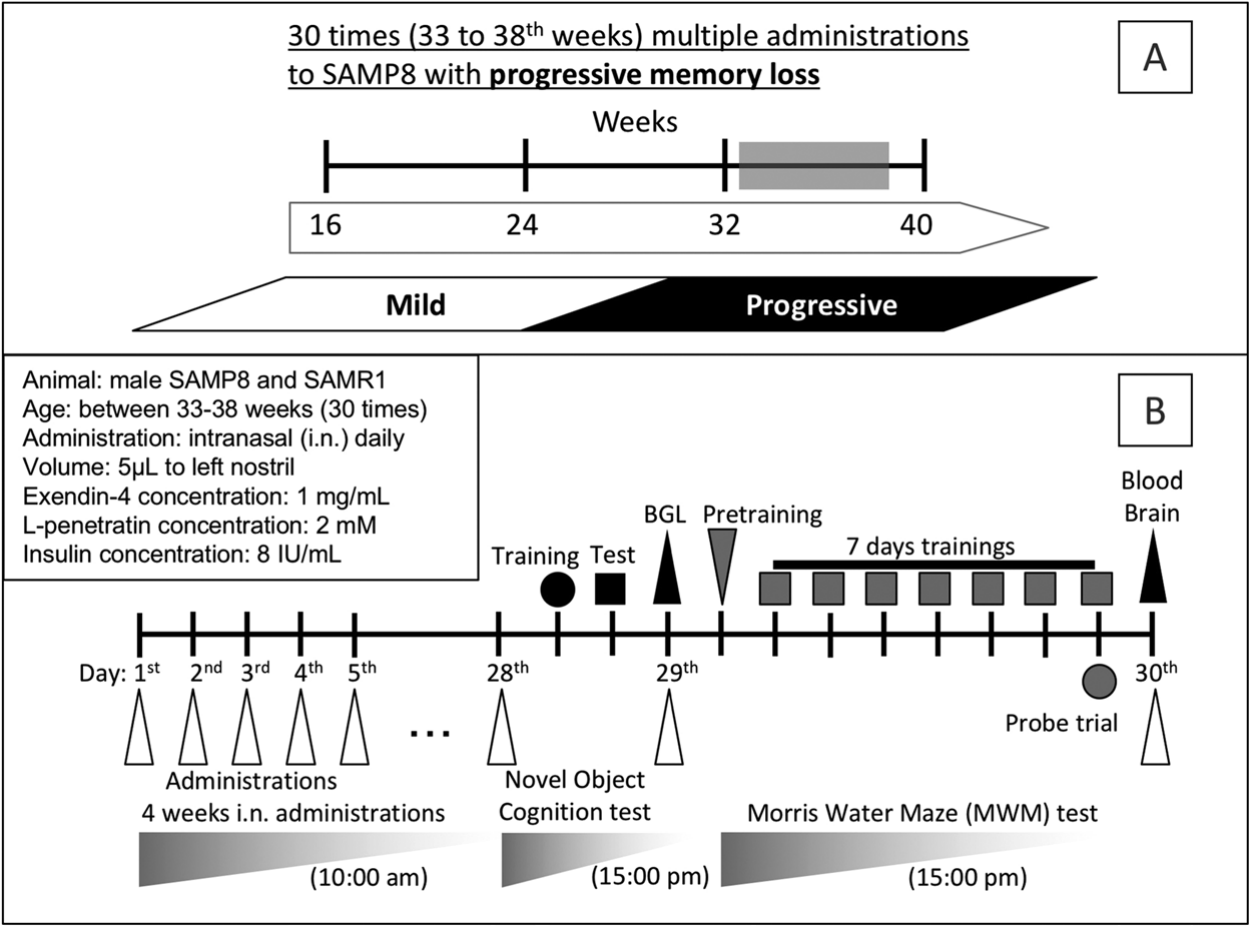
Schematic diagram of the study conducted to examine the therapeutic potential of nose-to-brain exendin-4 delivery boosted by coadministration with CPP (penetratin) in the mouse model of dementia. Panel A definition of the mild and progressive stages of dementia in SAMP8^22^. Panel B experimental protocols for examining the effects of exendin-4 delivery on progressive cognitive dysfunction.

Thus, the strategy to potentially target an Aβ-related mechanism may not be effective for the treatment of dementia at the severe stage, as demonstrated by the inefficient effect of antibody drugs via Aβ modulation^1,5^ and the increase in Aβ accumulation with excess insulin^22^. We hypothesized that rather than Aβ modulation, the strategy to stimulate insulin signaling with high specificity in the brain can improve progressive cognitive dysfunction in severe dementia. In the present study, we focused on the use of GLP-1 receptor agonists as the enhancer for insulin signaling in the hippocampal and cerebral neuronal cells without accelerating Aβ depositions. In addition, incretins such as GLP-1 pose a lower risk of hypoglycemia upon excess delivery to systemic circulation via coadministration with CPPs. Thus, we aimed to deliver GLP-1 and its analog (exendin-4) to the brain via the nose-to-brain delivery system by coadministration with the CPP penetratin. Furthermore, the activation of insulin-signaling pathway via the enhanced delivery of exendin-4 and its contribution to the therapeutic effect on the progressive memory loss of aged SAMP8 (33–38 weeks) were examined according to the scheme shown in Fig. 1.

## Results

### Effect of penetratin on the nose-to-brain delivery of GLP-1 and its analog in ddY mice

**Figure 2:**
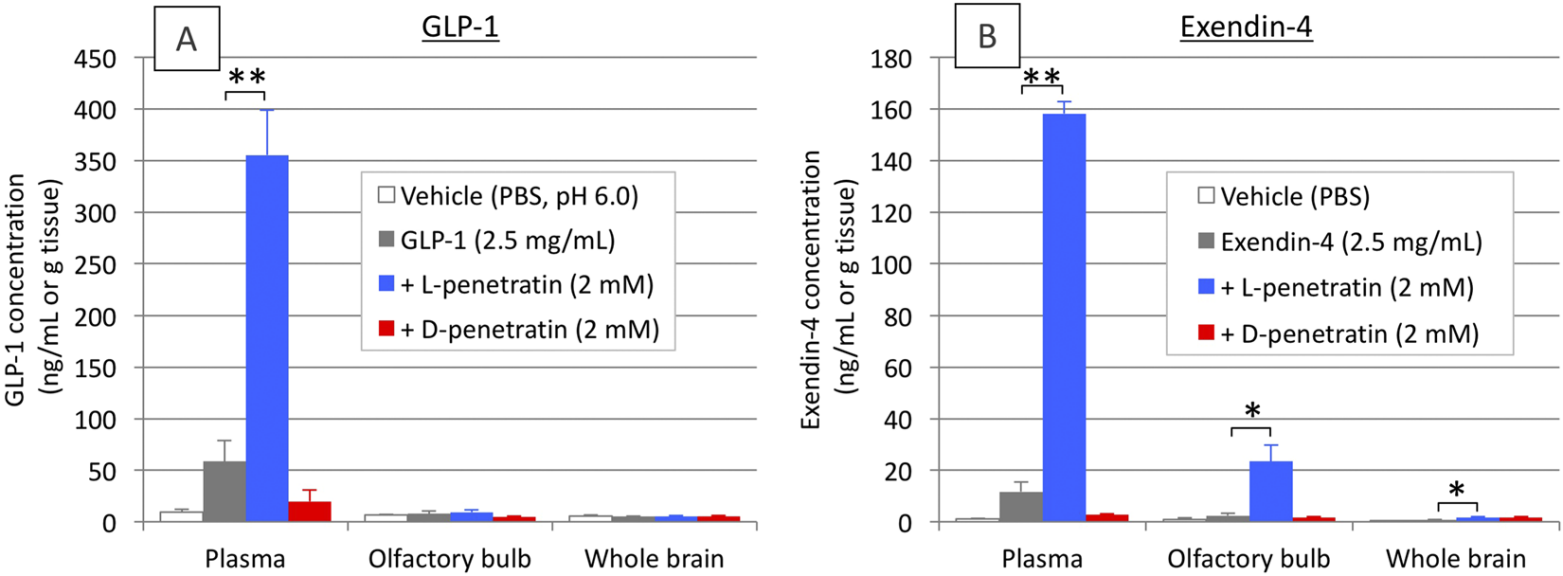
Systemic absorption and brain transport of GLP-1 and its analog exendin-4, following single intranasal administration of GLP-1 (2.5 mg/mL = 0.625 mg/kg) and exendin-4 (2.5 mg/mL = 0.625 mg/kg) with or without L- or D-penetratin (2.0 mM) to male ddY mice. Panels A and B show the results after intranasal administration of GLP-1 (30 min) and exendin-4 (15 min), respectively. Each data point represents the mean ± SEM of n = 3–4. *p < 0.05, **p < 0.01 indicate significant difference from the control group receiving GLP-1 or exendin-4 solution.

In the present study, we used GLP-1 and its analog exendin-4 as potential candidates for the treatment of progressive cognitive dysfunction in dementia such as AD. First, we examined the ability of the amphipathic CPP penetratin to facilitate the nose-to-brain delivery of GLP-1 and exendin-4 (both 2.5 mg/mL) in normal ddY mice. As shown in Fig. 2, intranasal coadministration with L-penetratin (2 mM) significantly enhanced the systemic absorption of both GLP-1 and exendin-4. Moreover, the concentration of exendin-4 in the olfactory bulb at 15 min post administration was significantly increased by coadministration with L-penetratin (Fig. 2B), although that of GLP-1 was not affected (Fig. 2A). The distribution of exendin-4 throughout the brain was also increased by coadministration with L-penetratin (control, 0.74 ± 0.10 ng/g tissue vs. with L-penetratin, 1.71 ± 0.33 ng/g tissue), although the absolute concentration in the whole brain was much lower than that in the olfactory bulb (Fig. 2B). In contrast to the effect of L-penetratin, D-penetratin could not enhance the systemic and brain delivery of GLP-1 and exendin-4 after nasal administration.

### Effect of penetratin on the brain distribution of exendin-4 after its intranasal administration

**Figure 3:**
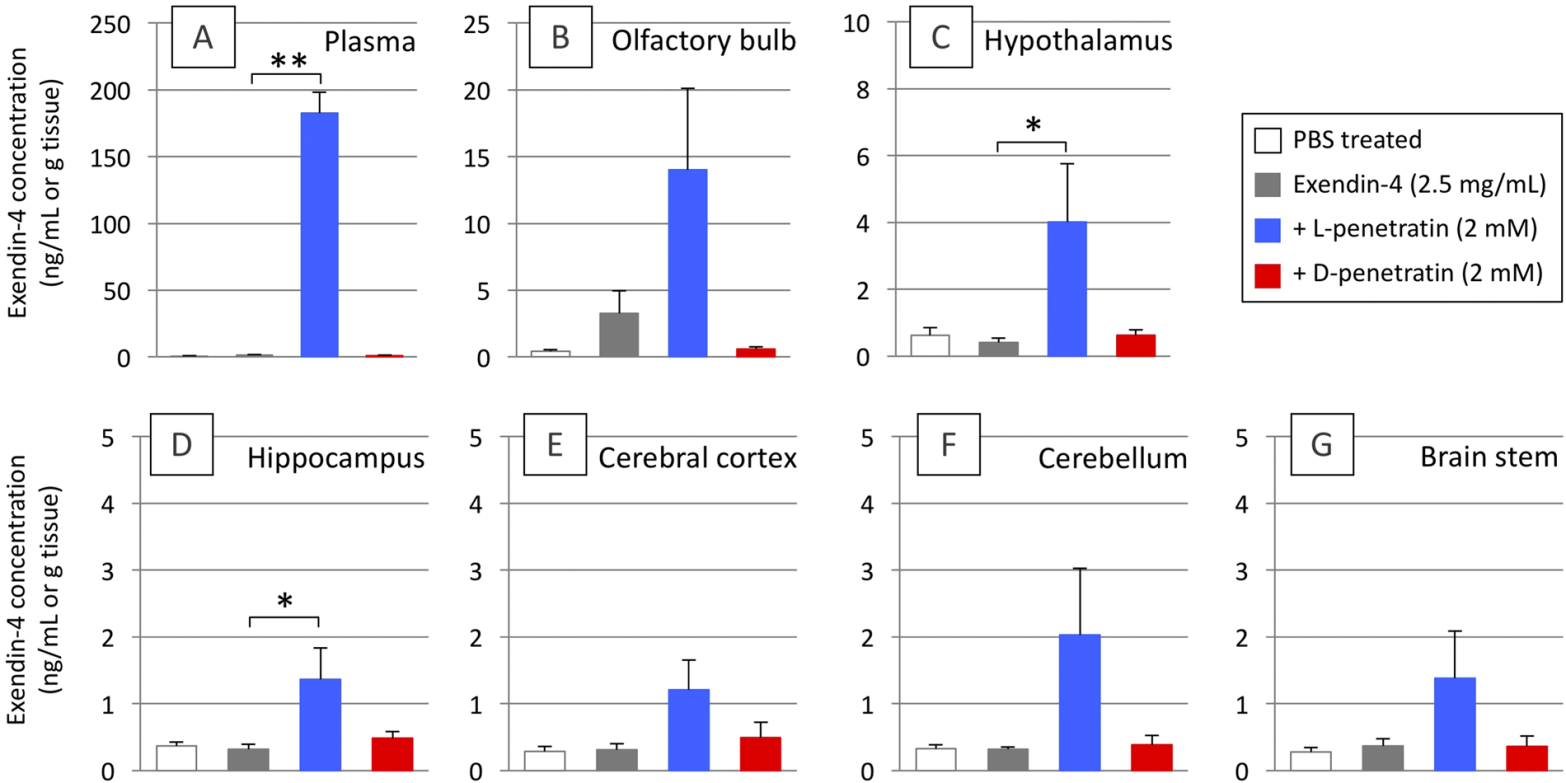
Distribution of exendin-4 in the brain, 15 min after a single intranasal administration of exendin-4 (2.5 mg/mL) with L- or D-penetratin (2 mM) to male ddY mice. Panels show exendin-4 levels in the plasma (**A**), olfactory bulb (**B**), hypothalamus (**C**), hippocampus (**D**), cerebral cortex (**E**), cerebellum (**F**), and brain stem (**G**). Columns show the results of intranasal exendin-4 administration in the absence (gray) or presence of L-penetratin (blue) or D-penetratin (red) and treatment with PBS as a vehicle (white). Each data points represents the mean ± SEM of n = 3–4. *p < 0.05, **p < 0.01 indicate significant difference from the control group receiving exendin-4 solution.

The above-mentioned results suggested that L-penetratin, but not D-penetratin, is a useful carrier in facilitating the delivery of exendin-4 to the brain, which is the primary site for cognitive dysfunction in AD. Therefore, we further examined the distribution of exendin-4 into the six parts of the brain after its intranasal coadministration with L- and D-penetratin. As shown in Fig. 3, the concentrations of exendin-4 in all six parts (olfactory bulb, hypothalamus, hippocampus, cerebral cortex, cerebellum, and brain stem) increased after intranasal coadministration with L-penetratin (2 mM), compared with that observed when it was administered without penetratin (Fig. 3B–G). The increase in exendin-4 levels was particularly significant in the hippocampus, where exendin-4 acts as the mediator for memory and learning (Fig. 3D). The results suggested that L-penetratin is a potential enhancer, facilitating nose-to-brain delivery for the treatment of dementia.

### Activation of insulin signaling-related protein via enhanced nose-to-brain delivery of exendin-4

**Figure 4:**
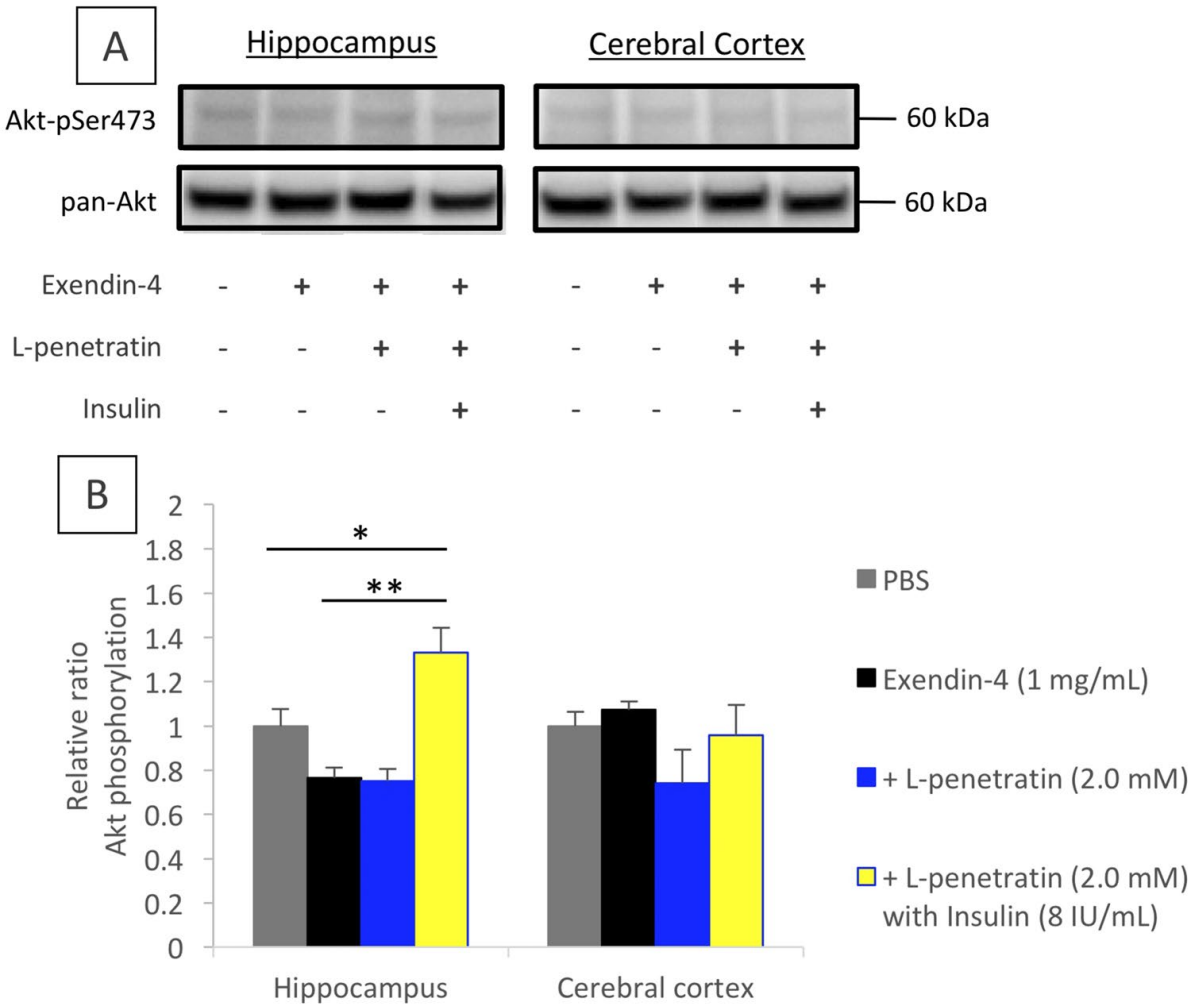
Analysis of the phosphorylation of Akt in the brain after single intranasal administration of exendin-4 with L-penetratin and insulin to male ddY mice. Panel A shows the typical band detected by immunoblotting with anti-Akt-pSer473 or anti-pan-Akt antibodies. The full-length blots from the same experimental gel are presented in Supplementary Figure (Fig. S1). Panel B shows the quantitated Akt activation corrected by pan-Akt levels. Each data point represents the mean ± SEM of n = 3. *p < 0.05, **p < 0.01 indicate significant difference between the groups.

GLP-1 receptor agonists such as exendin-4 have been reported to promote the activation of insulin signaling in the brain by stimulating GLP-1 receptor, which facilitates the downstream molecule, insulin receptor substrate (IRS)−1^11^. Therefore, exendin-4 is an ideal candidate to correct the severe condition in which the insulin receptor is lost from the cell membrane by the action of a soluble Aβ oligomer^11^. We examined whether efficient delivery of exendin-4 to the hippocampus and cerebral cortex by intranasal coadministration with L-penetratin to ddY mice could be linked to insulin signaling via detection of the phosphorylation of Akt (protein kinase B), a downstream molecule in the insulin-signaling pathway. Figure 4A shows the typical band patterns after immunoblotting for phosphorylated and total Akt after intranasal administration of exendin-4 with or without L-penetratin and insulin, and the relative ratio of Akt phosphorylation was calculated as shown in Fig. 4B. The results revealed that the simultaneous delivery of exendin-4 and insulin to the brain via intranasal administration with L-penetratin could contribute to insulin signaling in the hippocampus (Fig. 4B). Importantly, the results further suggested that the nose-to-brain delivery of exendin-4 via coadministration with L-penetratin could not facilitate insulin signaling without primarily stimulating the insulin receptor via adequate levels of insulin in the brain. In this examination, a relatively low dose of insulin (8 IU/mL) was coadministered with exendin-4 and L-penetratin, as our previous study suggested that excess levels of insulin might potentially induce an elevation in Aβ^22^. Although the insulin treatment group without exendin-4 was not included in this examination, insulin signaling in the brain might be partially activated by delivery of only insulin in ddY mice.

### Therapeutic effect of nose-to-brain exendin-4 delivery facilitated by coadministration with L-penetratin for progressive memory loss in SAMP8

**Figure 5:**
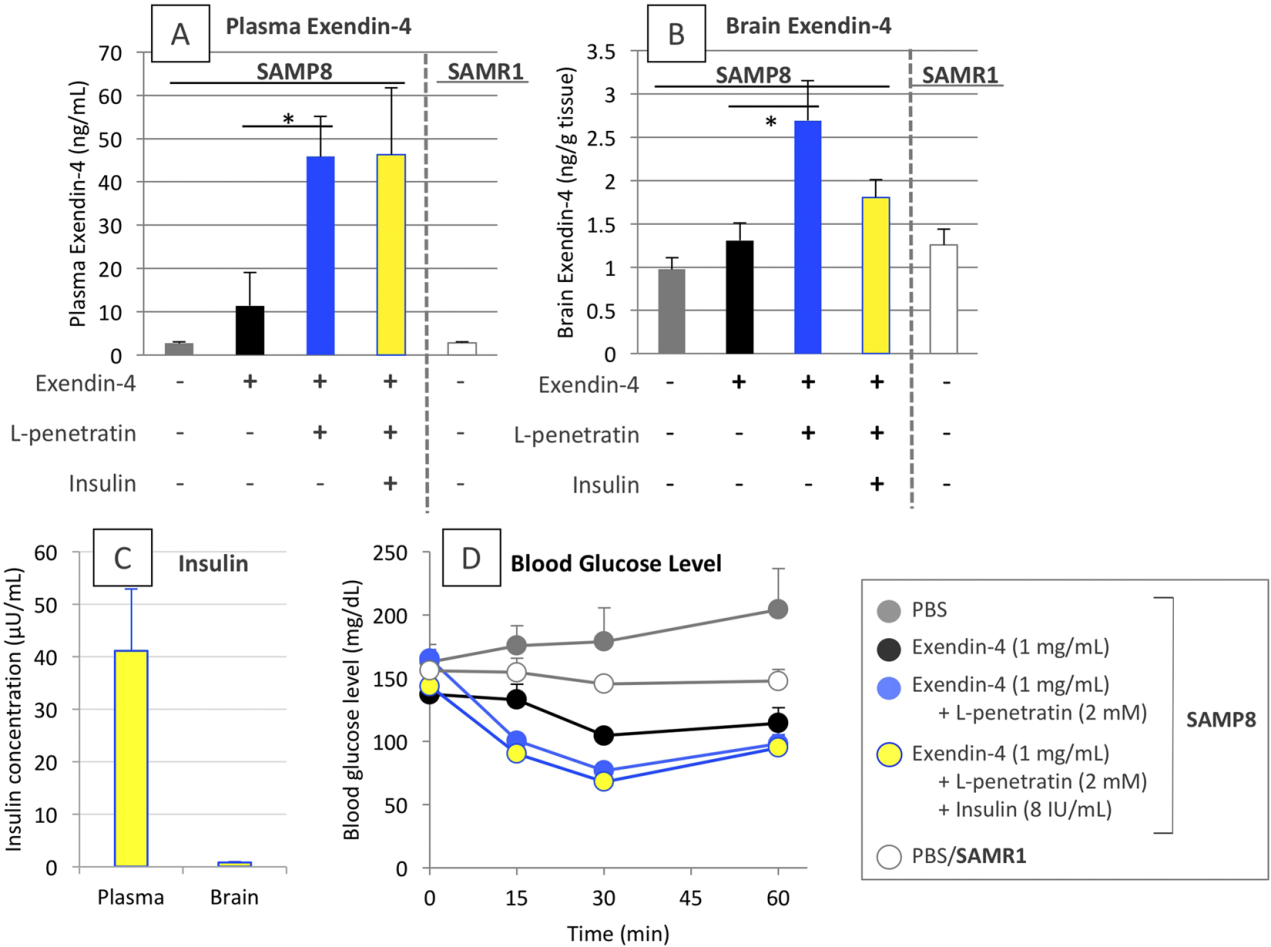
Systemic absorption and brain distribution of exendin-4 and insulin, and the subsequent hypoglycemic effect after long-term multiple administrations of exendin-4 with or without L-penetratin and additionally with insulin, to SAMP8 mice with progressive cognitive dysfunction. Panels A and B exendin-4 concentration in the plasma and the brain, respectively, at 15 min after the 30^th^ administration. Panel C insulin concentration in the plasma and brain of mice intranasally receiving exendin-4, L-penetratin, and insulin at 15 min after the 30^th^ administration. The concentration in mice not receiving insulin was not measured because of no cross reactivity between administered human insulin and endogenous mouse insulin in ELISA. Panel D time-dependent changes in the blood glucose levels after the 29^th^ administration. Tests were conducted at 37 (blood glucose) or 38 (plasma exendin-4 and insulin) weeks of age. Each data point represents the mean ± SEM of n = 4–8. *p < 0.05 indicates significant difference from SAMP8 receiving exendin-4 solution.

The above-mentioned results based on the pharmacokinetic and biological examinations suggested that exendin-4 delivered to the brain via coadministration with L-penetratin could contribute to improvements in memory and learning, in concert with insulin. Therefore, we next examined the therapeutic effect of the facilitated nose-to-brain delivery of exendin-4 by coadministration with L-penetratin. In this examination, SAMP8 and SAMR1 (33–38 weeks old) were used as dementia model mice with progressive cognitive dysfunction and normal (control) mice, respectively, which were repeatedly administered exendin-4 solution (1 mg/mL) containing L-penetratin (2 mM) with or without insulin (8 IU/mL) for 30 days. On the last day of long-term intranasal administrations, we confirmed that the concentrations of exendin-4 in the plasma and whole brain increased after coadministration with L-penetratin to SAMP8, in the presence and absence of insulin (Fig. 5A,B). In contrast, the brain concentration of insulin was less than the lower detection limit because the dose of insulin should be kept low for avoiding Aβ accumulation by its excess delivery (Fig. 5C)^22^. However, the relatively high level of insulin in plasma (Fig. 5C, 41.1 ± 11.8 μU/mL) confirmed that insulin was also supplied adequately to the brain after coadministration with L-penetratin. Blood glucose was maintained at safe levels (>70 mg/dL), reducing the risk of hypoglycemia, even after exendin-4 and insulin were systemically absorbed by coadministration with L-penetratin (Fig. 5D).

**Figure 6:**
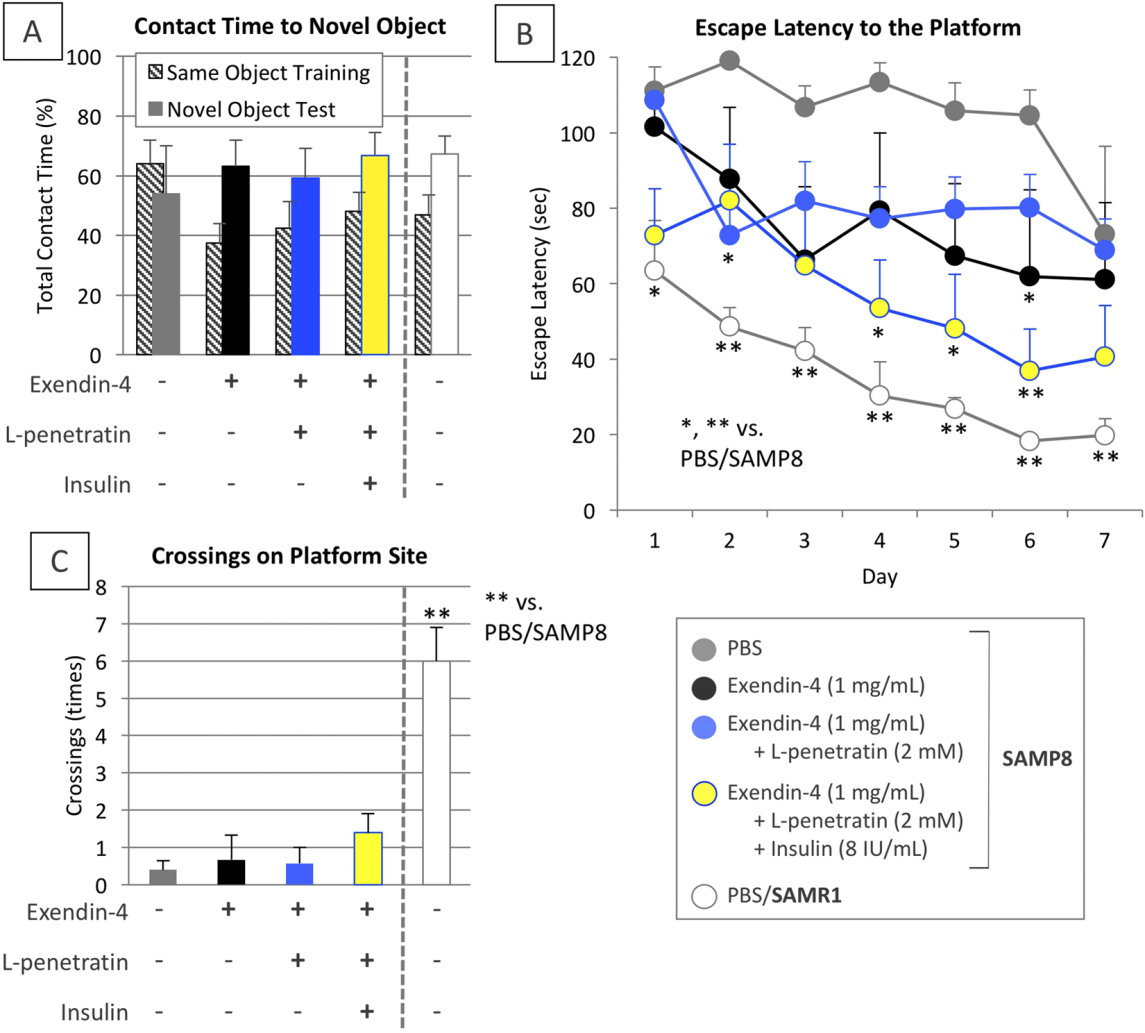
Scores in the cognitive learning tests after long-term (30 times) repeated intranasal administrations to SAMR1 control and SAMP8 with progressive cognitive dysfunction. Panel A ratios of the contact time to original and novel objects during the NOR test. Panels B,C changes in the escape latency to the platform as an index of cognitive learning during the MWM test and crossing numbers across the platform area after its removal in the probe trial, respectively. Both tests were conducted at 37–38 weeks of age. Each data point represents the mean ± SEM of n = 4–8. *p < 0.05, **p < 0.01 indicate significant difference from SAMP8 receiving PBS.

To test the therapeutic effect of exendin-4 delivered to the brain on cognitive dysfunction in the progressive stage, two behavioral tests were conducted. The results in the novel object recognition (NOR) test (Fig. 6A) showed that the SAMP8, without receiving any treatment, were equally interested in both original and novel objects, confirming memory loss. In contrast, the SAMP8 receiving intranasal administration of exendin-4 even without L-penetratin and insulin tended to curiously assess the novel object, suggesting that the slight accumulation of exendin-4 in the brain after intranasal administration without L-penetratin might contribute to learning in the SAMP8 at the progressive stage. Moreover, the escape latency scores in the Morris water maze (MWM) test (Fig. 6B) demonstrated that intranasal exendin-4 administration without L-penetratin, and coadministration of L-penetratin (2 mM) and insulin (8 IU/mL) further boosted the therapeutic effect of exendin-4 by a combined effect of enhanced nose-to-brain delivery of exendin-4 and of activation of the main route for insulin signaling (Fig. 6B). These effects were not examined in SAMP8. The crossing scores in the probe trial on the day following the end of training period also suggested that intranasal coadministration of exendin-4 with L-penetratin with insulin was most effective for improving memory among the test groups, although the score of SAMP8 receiving the triple administrations could not be achieved in normal SAMR1 (Fig. 6C).

### Effect of nose-to-brain exendin-4 delivery with L-penetratin on neurodegeneration and Aβ accumulation in the brain

The above-mentioned results suggested that effective delivery of exendin-4 and adequate supplementation of insulin in the brain via intranasal administration is a promising approach for treating dementia with severe cognitive dysfunction. Considering the pathological and pharmacological mechanisms for dementia such as AD, this successful observation might be achieved by three factors, (1) recovery of neurodegeneration, (2) reduction in the cerebral and hippocampal Aβ levels in soluble and insoluble forms, and (3) activation of insulin signaling via the stimulation of insulin and GLP-1 receptors.

**Figure 7:**
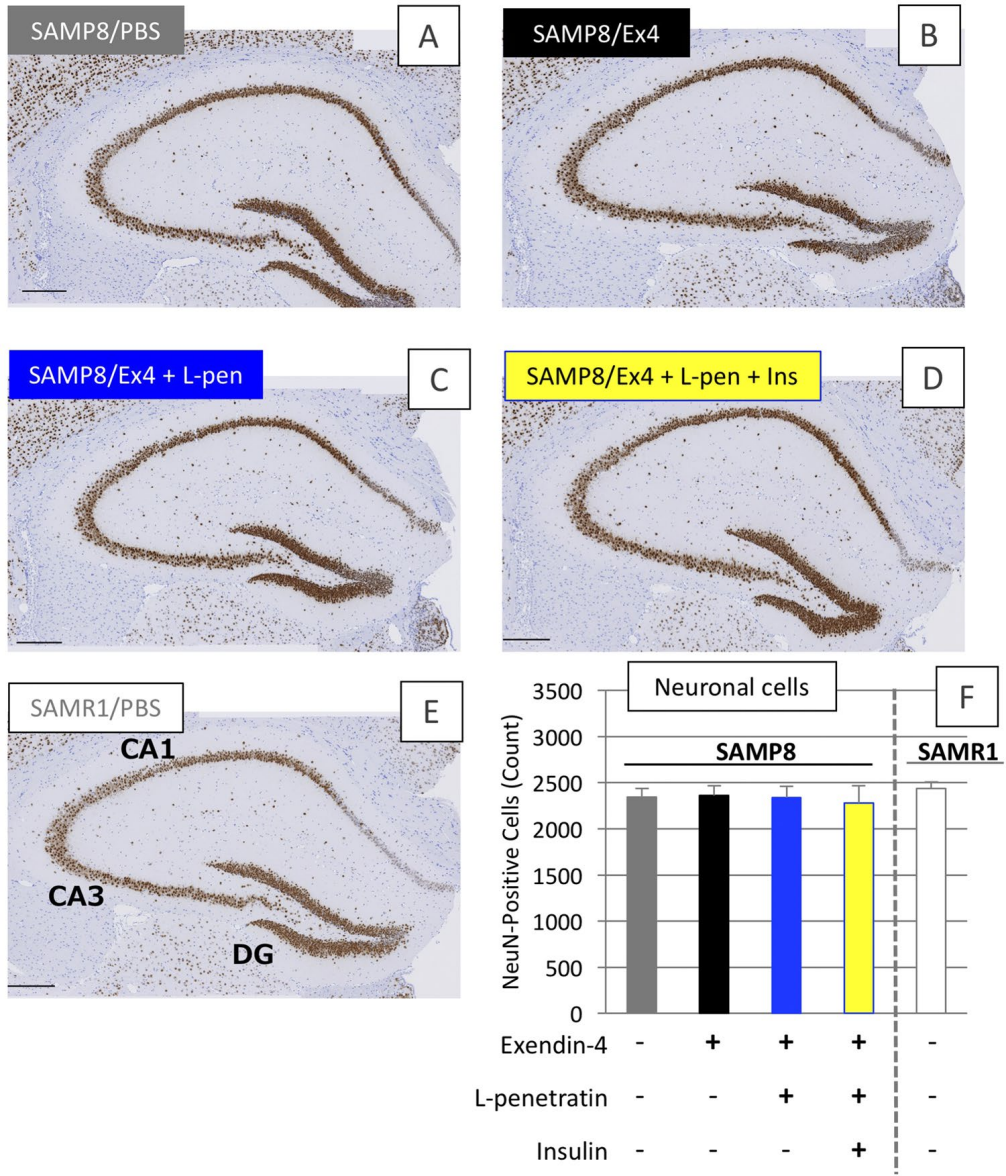
Immunohistological staining of brain specimens with anti-NeuN antibody after long-term (30 times) intranasal administrations to SAMR1 control and SAMP8 with progressive cognitive dysfunction. (**A**–**E**) Microscopic observations of hippocampal areas of SAMP8 administered PBS (N = 5), exendin-4 only (N = 4), exendin-4 with L-penetratin (N = 7), or exendin-4 with L-penetratin plus insulin (N = 5) and SAMR1 administered PBS (N = 8), respectively. Panel F quantitative analysis of the NeuN-positive cells calculated from each image. CA, cornu ammonis, DG, dentate gyrus. Tests were conducted at 38 weeks of age. Each data point represents the mean ± SEM of n = 4–8.

Figure. 7A–E shows the immunohistological staining of the coronal slices of brain specimens with anti-NeuN antibody to estimate the neuronal cell condition after the 30^th^ intranasal administration of exendin-4 in the presence or absence of L-penetratin with or without insulin (Fig. 1). As shown in Fig. 7F, the neuronal cell counts estimated from the images (Fig. 7A–E) clarified that there is no difference between normal SAMR1 and disordered SAMP8, and that exendin-4 and insulin delivered to the brain after intranasal administration had no proliferation effect on the hippocampal neuronal cells.

**Figure 8:**
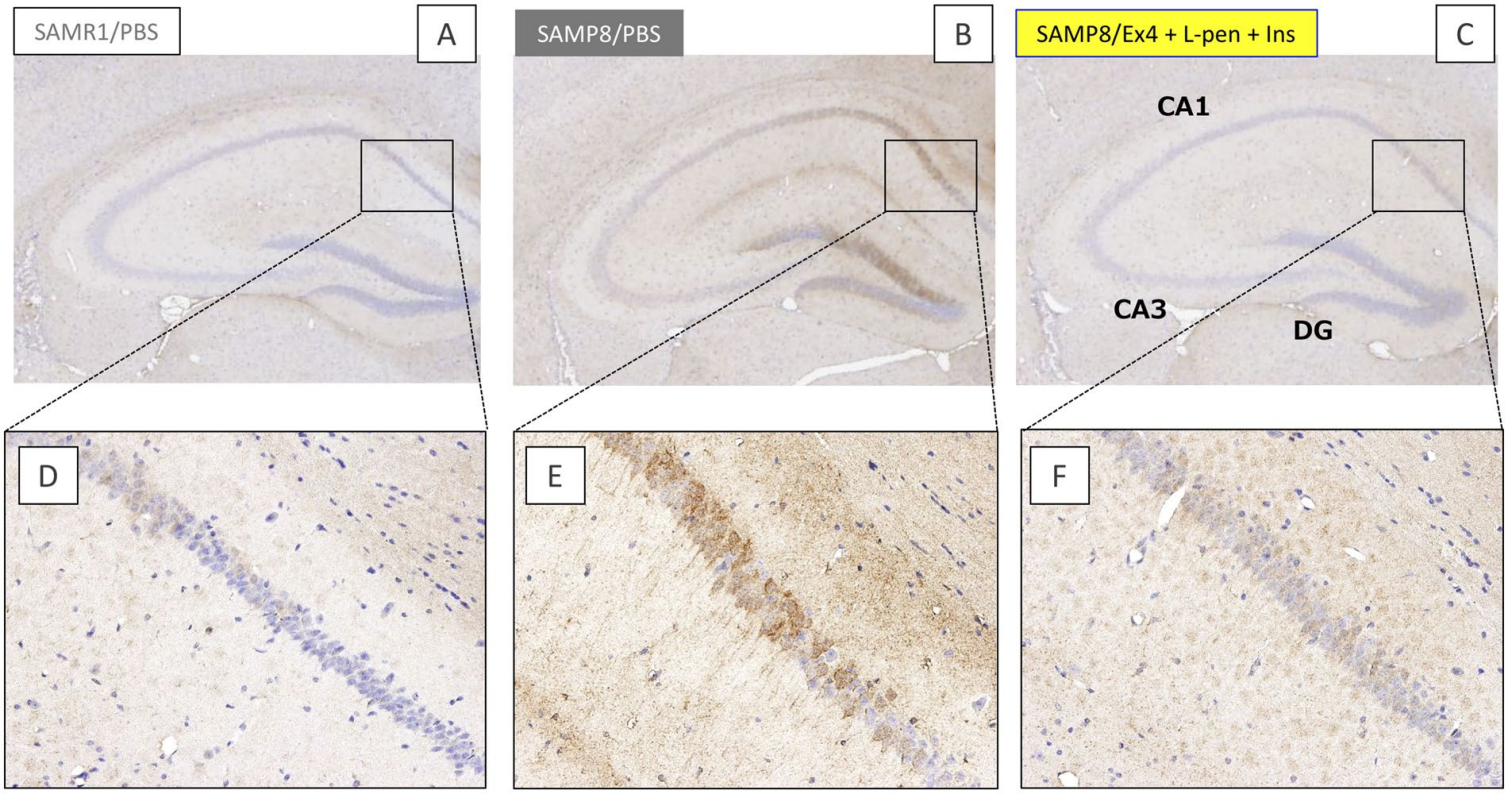
Immunological staining of the brain specimens with anti-human Aβ (1–42) antibody after the long-term (30 times) intranasal administration to SAMR1 control and SAMP8 with progressive cognitive dysfunction. (**A**–**C**) Microscopic observations of hippocampal areas of SAMR1 administered PBS (N = 8) and those of SAMP8 administered PBS (N = 5) or exendin-4 with L-penetratin plus insulin (N = 5). (**D**–**F**) Enlarged images of the areas indicated in panels A–C, respectively. CA, cornu ammonis, DG, dentate gyrus. Tests were conducted at 38 weeks of age.

**Figure 9:**
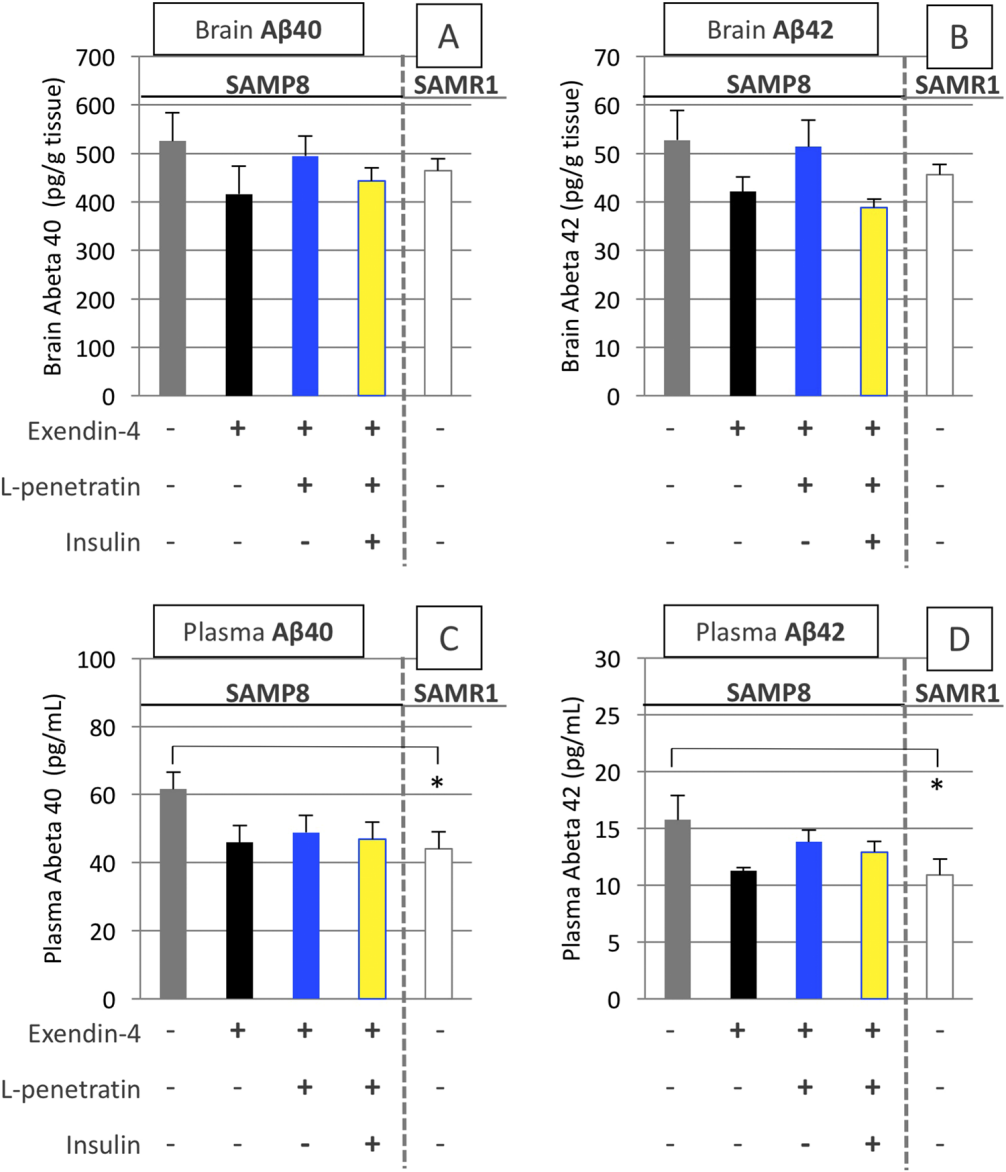
Soluble Aβ (1–40) and Aβ (1–42) levels in plasma and brain after long-term (30 times) intranasal administration to SAMR1 control and SAMP8 with progressive cognitive dysfunction. Panels A,B soluble Aβ (1–40) and Aβ (1–42) in brain, respectively. Panels C and D soluble Aβ (1–40) and Aβ (1–42) in plasma, respectively. Tests were conducted at 38 weeks of age. Each data point represents the mean ± SEM of n = 4–8. *p < 0.05 indicates significant difference from SAMP8 receiving PBS.

Our previous work suggested that excess delivery of exogenous insulin into the brain potentially increases the quantity of Aβ plaque in the hippocampus of SAMP8 with progressive cognitive dysfunction^22^. Therefore, we next examined the effect of exendin-4 and insulin, delivered efficiently and adequately to the brain via intranasal administration, on the Aβ depositions in the brain of SAMP8 mice. As shown in Fig. 8B,E, staining with the anti-Aβ (1–42) antibody was highly concentrated in the hippocampus of SAMP8 after the administration of vehicle (phosphate-buffered saline, PBS), but there were no remarkable plaques of Aβ consistent with our previous study^22^. In contrast, the hippocampus of SAMP8 receiving intranasal exendin-4 with L-penetratin and insulin (Fig. 8C,F) was lightly stained, comparable with the normal SAMR1 receiving PBS (Fig. 8A,D). The quantification of soluble Aβ (1–40) and Aβ (1–42) in the brain by ELISA (Fig. 9A,B, respectively) showed a small difference between the groups. This might suggest that nasal administration of exendin-4 has the potential to reduce the accumulation of soluble Aβ in the brain, even without L-penetratin and supplemental insulin stimulation.

On the other hand, plasma level of Aβ may be used as an index to estimate the progress of dementia. As shown in Fig. 9C,D, the increased accumulation of Aβ (both subtypes, Aβ (1–40) and Aβ (1–42)) was reduced by intranasal treatment with exendin-4, regardless of coadministration of L-penetratin with or without insulin. Thus, the results suggested that the partial amelioration of progressive cognitive dysfunction in SAMP8 by intranasal administration of exendin-4 without L-penetratin and insulin (Fig. 6A,B) could be attributed to the reduction of Aβ in plasma and the brain. Additionally, as shown in Fig. 4 in which hippocampal Akt phosphorylation was facilitated in ddY mice, the activation of insulin signaling in the hippocampus after administration of exendin-4 with L-penetratin and adequate insulin could possibly achieve further stimulation of learning and memory in SAMP8 (Fig. 6B,C).

## Discussion

The therapeutic target for dementia is the brain, which is protected by the BBB as mentioned above. To deliver macromolecular drugs such as insulin across the BBB, several approaches using chemical conjugates with endogenic ligands and tight junction modulators have been tried to date, however, none have been established as of yet^26^. Alternatively, intranasal administration has been studied and recognized as the ideal route for directly delivering drugs to the central nervous system by bypassing the BBB, and some studies have shown its potential contribution to the pharmacotherapy of various diseases and disorders such as AD, autism, and brain cancer^8,27,28^. On the other hand, our previous study clarified that the absolute amount of macromolecular drugs that can reach the brain via intranasal administration is relatively low, because of inefficient uptake and permeation through the nasal epithelium, particularly in the olfactory region^20^.

Our recent study demonstrated that efficient delivery of insulin to the brain via its intranasal administration with L-penetratin, which is the most effective CPP for transepithelial delivery of biopharmaceuticals^29^, could prevent the progression of mild cognitive dysfunction in SAMP8 as model dementia mice^22^. In the same study, however, this strategy failed to counteract progressive cognitive dysfunction in aged SAMP8^22^. The immunohistological analysis of the brain with anti-Aβ antibody suggested that insulin delivered in excess to the brain after intranasal coadministration with L-penetratin could be preferentially recognized by IDE, which also acts as a potent enzyme contributing to the clearance of Aβ^23–25^. Owing to the competition for IDE with excess insulin, Aβ might not be degraded by IDE, and therefore, it was unexpectedly accumulated in the brain. The nose-to-brain delivery of insulin is a promising strategy for preventing memory loss, however its therapeutic potential is limited to the early stage of dementia.

To overcome the difficulty of therapy via nose-to-brain delivery of insulin in the progressive stage of dementia, we focused on the potential of GLP-1 and its analogs as alternative drug candidates. The GLP-1 receptor agonists have been shown to play a role in memory and learning in the brain by stimulating their associated receptor and further mediating the downstream processes of insulin signaling^11^. Therefore, we first tried to deliver GLP-1 and its analog exendin-4 (Exenatide, as a synthetic peptide) to the brain via intranasal administration with penetratin. As shown in Fig. 2A, the coadministration with L-penetratin could facilitate the systemic absorption of both GLP-1 and exendin-4. L-penetratin could also enhance the delivery of exendin-4 from the nasal cavity to the olfactory bulb, which is the gate of nose-to-brain delivery^18,19^, and eventually exendin-4 was distributed throughout the brain (Fig. 2B). In contrast, GLP-1 could not effectively reach the brain through intranasal administration with L-penetratin (Fig. 2A), uncorrelated to its increased systemic absorption. This suggested that the enhancement of systemic drug absorption does not always contribute to an increase in the drug concentration in the brain, and that increase in the brain level of exendin-4 after intranasal coadministration with L-penetratin might be achieved by facilitating its direct transport from nasal cavity to the brain. On the other hand, the proteolytically stable D-penetratin could not increase the systemic absorption and brain delivery of these peptide drugs (Fig. 2 A,B), because the more stable drug complexes formed with D-penetratin might remain in the cytoplasm as compared to the less stable L-penetratin-drug complexes^29,30^. This would result in less release to the lamina propria, which is the middle point of the olfactory bulb^21^. Further examination of the distribution of exendin-4 in the brain confirmed that upon intranasal coadministration with L-penetratin, exendin-4 was delivered to the all parts of the brain separately, including the olfactory bulb, hypothalamus, hippocampus, cerebral cortex, and cerebellum (Fig. 3). Thus, L-penetratin can be a useful carrier peptide for delivering exendin-4 via the nose-to-brain direct transport pathway.

The pharmacological examinations based on the NOR and MWM tests demonstrated that the multiple nasal administration of exendin-4 even without L-penetratin for 4 weeks could partially improve the progressive cognitive dysfunction of SAMP8 (Fig. 6A,B). Akt phosphorylation downstream of insulin signaling in the hippocampus could not be facilitated in ddY mice by intranasal administration of exendin-4 without L-penetratin and insulin (Fig. 4). This suggests that the partial improvement of progressive cognitive dysfunction in SAMP8 after administration of exendin-4 without L-penetratin and insulin (Fig. 6A,B) was mediated by a mechanism different from insulin signaling activation. On the other hand, the simultaneous nose-to-brain delivery of exendin-4 and insulin together with coadministration of L-penetratin could additionally improve spatial cognitive function, as shown in the MWM test (Fig. 6B,C). This additional improvement of progressive cognitive dysfunction mediated by exendin-4 and insulin might be associated with the activation of insulin signaling, as shown in Fig. 4B. Presumably, the appropriate amount of insulin, which does not affect Aβ deposition, might stimulate its receptors to be limitedly expressed on the hippocampal neuronal cell surface under pathological conditions^31^. Subsequently, insulin signaling initiation might be further boosted by the stimulation of GLP-1 receptor on the neuronal cells with exendin-4, through the activation of downstream molecule IRS-1^11^. Throughout the present study, insulin was added to exendin-4 and L-penetratin mixed solution at its supplemental dose (1 IU/kg), which is lower than the dose used in our previous study (5 IU/kg), as excess nose-to-brain delivery of insulin by the coadministration of L-penetratin might unexpectedly develop Aβ accumulation in the brain^22^.

In the present study, we found that efficient nose-to-brain delivery of exendin-4 and supplementation of insulin at adequate doses could contribute to the reversal of the severe cognitive dysfunction in the SAMP8. However, based solely on the present study, we could not provide the evidence that brain delivery of exendin-4 and insulin has the potential to repair the neuronal cells degenerated by Aβ oligomers or plaques, because the examinations were conducted on senescence-accelerated model mice, without a significant degeneration of neuronal cells (Fig. 7F). In our recent study, we reported that intranasal insulin administration showed the potential to prevent neurodegeneration in aged SAMP8 with progressive cognitive dysfunction. However, no difference in the neuronal cell numbers was observed between SAMP8 and SAMR1 in the present study, as the induction of neurodegeneration might be varied depending on environmental factors such as cleanness in the room and resultant stress. By reexamining the effect of intranasal exendin-4 delivery in SAMP8 with neurodegeneration, the therapeutic potential of exendin-4 and/or insulin via intranasal administration could be more precisely evaluated. On the other hand, our data suggest that Aβ accumulated in the brain and plasma might be cleared by the intranasal administration of exendin-4, regardless of the delivery efficiency to the brain (Figs 8 and 9). However, unlike the conventional transgenic mice with amyloid precursor protein (APP)/presenilin-1 (PS1)^32^, the SAMP8 used in this study likely had marginal levels of Aβ plaque. Thus, based solely on the present study, we cannot solidly establish the potential of nose-to-brain delivery of exendin-4 as a therapeutic strategy for AD, because the characteristics of SAMP8 are different from the pathology of AD patients. However, the fact that exendin-4 and insulin effectively and adequately delivered to the brain via intranasal administration have the potential to activate insulin signaling (Fig. 4) and improve severe cognitive dysfunction (Fig. 6) showed the advantage of our strategy in being a versatile method for treating various types of dementia, including Lewy body- and vascular-types and frailty, via an Aβ-independent pathway. In future studies, we would attempt to show that effective nose-to-brain delivery of exendin-4 via coadministration with CPPs is a promising strategy for the treatment of AD by using several types of animal models for brain pathology.

## Methods

### Materials

Exendin-4 (HGEGTFTSDLSKQMEEEAVRLFIEWLKNGGPSSGAPPPS-NH_2_, 83.8%, 4189.7 Da), GLP-1 (HAEGTFTSDVSSYLEGQAAKEFIAWLVKGR-NH_2_, 89.2%, 3299.9 Da), l-Penetratin (RQIKIWFQNRRMKWKK; capital letters indicate the l-form of amino acids, >95%, 2247.3 Da) and d-penetratin (rqikiwfqnrrmkwkk; lowercase letters indicate the d-form of amino acids, >95%, 2245.8 Da) were synthesized by Sigma-Genosys, Life Science Division of Sigma-Aldrich Japan Co. (Hokkaido, Japan). Recombinant human insulin (27.5 IU/mg), GLP-1 ELISA kit, and 4% paraformaldehyde in phosphate buffer (PFA) were purchased from Wako Pure Chemical Industries, Ltd. (Osaka, Japan). *p*-Chloromercuribenzoic acid (PCMB), anti-phospho-Akt (pSer473) antibody (SAB4504331), and pan-Akt antibody (SAB4301170) were purchased from Sigma-Aldrich Co. (Darmstadt, Germany). Complete™ protease inhibitor cocktail tablets were purchased from Roche Ltd. (Basel, Switzerland). EzRIPA Lysis kit was purchased from ATTO Corp. (Tokyo, Japan). Anti-NeuN rabbit monoclonal antibody (EPR12763) and goat anti-rabbit IgG H&L (HRP) (ab6721) were purchased from Abcam plc (Cambridge, UK). Anti-human Aβ (1–42) rabbit IgG (code #18582), mouse/rat Aβ (1–40) assay kit (code #27720), and mouse/rat Aβ (1–42) assay kit (code #27721) were purchased from (Immuno-Biological Laboratories Co. Ltd., Gunma, Japan). Exendin-4 EIA kit was purchased from Phoenix Pharmaceuticals, Inc., (Burlingame, CA, USA). Insulin human ELISA kit and insulin human ELISA ultrasensitive kit were purchased from Mercodia AB (Uppsala, Sweden). Methylcellurose (MC, METOLOSE) was purchased from Shin-Etsu Chemical Co., Ltd. (Tokyo, Japan). All other chemicals were commercially available and of analytical grade.

### Animals

This study was performed at Kobe Gakuin University and complied with the regulations of the Committee on Ethics in the Care and Use of Laboratory Animals (approved as the Protocol number A14-34, A15-22, A16-15 and A17-04) and in accordance with the Related Activities in Academic Research Institutions under the jurisdiction of the Ministry of Education, Culture, Sports Science and Technology. Male ddY mice, SAMP8 (dementia model mice), and SAMR1 (normal control mice for SAMP8) were purchased from Japan SLC Inc. (Shizuoka, Japan). All mice were housed in rooms maintained under a 12-h light-dark cycle at 23 ± 1 °C and 55 ± 5% relative humidity, and were allowed free access to water and food during the acclimatization period.

### Preparation of mixed solutions of peptide drug and penetratin for single intranasal administration study

Specific amounts of peptide drug (GLP-1 or exendin-4) and penetratin (l- or d-penetratin) were dissolved separately in phosphate-buffered saline (PBS, pH 6.0) containing 0.001% methylcellulose (MC) as stock solutions (5 mg/mL and 4.0 mM, respectively), and equal volumes of peptide drug and penetratin solutions were mixed gently and adjusted to 2.5 mg/mL and 2.0 mM, respectively. Each GLP-1 or exendin-4 plus l- or d-penetratin solution was clear after mixing. For the western blot examination, the concentration of exendin-4 was adjusted to 1 mg/mL, and insulin (8 IU/mL) was further added to the exendin-4/L-penetratin solution.

### Single intranasal administration study with ddY mice

Mice (ddY, 7 weeks old) were anesthetized with an intraperitoneal (i.p.) injection of sodium pentobarbital (50 mg/kg; Somnopentyl, Kyoritsu Seiyaku Corp., Tokyo, Japan), and restrained in a supine position. A total of 10 μL (5 μL/nostril) of exendin-4 or GLP-1 solution with or without L- or D-penetratin (2.0 mM) was administered intranasally to the mice using a micropipette (Pipetman P-20, Gilson Inc., Middleton, WI, USA). Doses of both exendin-4 and GLP-1 were 0.625 mg/kg body weight (2.5 mg/mL). At 15 (exendin-4) or 30 (GLP-1) min post administration, 0.2 mL of blood was collected from the left jugular vein of the mice. The abdominal cavity was opened, and the intravascular content in the brain was flushed by a perfusion of ice-cold PBS (pH 7.4) into the left ventricle of the heart at 5.5 mL/min for 5 min using a peristaltic pump (ATTO Corp., Tokyo, Japan). The mice were quickly decapitated, and the whole brain was carefully isolated and washed with ice-cold PBS (pH 7.4). After removing the water content, the isolated brain sample was separated into two (olfactory bulbs and remaining portion) or six parts (olfactory bulbs, hypothalamus, hippocampus, cerebral cortex, cerebellum, and brain stem) on ice. These samples were weighed and homogenized with 4 times the volume of ice-cold assay buffer (Phoenix Pharmaceuticals, Inc.) using a glass or microtube tissue grinder. The blood sample and homogenized samples were centrifuged at 4 °C and 5,400 × *g* for 15 min, and the concentrations of exendin-4 and GLP-1 in the resultant plasma or homogenate supernatant were determined using an ELISA kit (exendin-4 EIA kit; Phoenix Pharmaceuticals, Inc., or GLP-1 ELISA kit; Wako Pure Chemicals Industries, Ltd.). In the study with GLP-1, the mice were fasted for 12 h before the intranasal administration, whereas this step was absent in the study with exendin-4, to avoid variation in the endogenous levels of GLP-1 in plasma and brain.

### Immunoblotting

Mice (ddY, 7 weeks old) were anesthetized with an i.p. injection of sodium pentobarbital and the exendin-4 solution with or without L-penetratin was intranasally administered as described above. Mice were administered 5 μL of exendin-4 solution (1.0 mg/mL) plus L-penetratin (2.0 mM) mixed solution through a micropipette (Pipetman P-20, Gilson, Inc., Middleton, WI, USA) inserted directly into their left nostril. The dose of exendin-4 was 0.125 mg/kg body weight (1.0 mg/mL). The additional group intranasally treated with the solution containing exendin-4, L-penetratin, and insulin (8 IU/mL) was also included in this study. The dose of insulin added to the exendin-4/L-penetratin solution was lower than that used in our previous study (1 IU/kg vs. 5 IU/kg) because an excess accumulation of insulin in the brain might unexpectedly increase the brain level of Aβ^22^. At 15 min post administration, the intravascular content in the brain was flushed as described above, and the whole brain was carefully isolated and washed with ice-cold PBS (pH 7.4). The isolated brain sample was separated into two parts (hippocampus and cerebral cortex) on ice. These samples were weighed and homogenized with 4 times the volume of ice-cold RIPA buffer containing protease and phosphatase inhibitor cocktail, using a glass or microtube tissue grinder. The homogenized samples were centrifuged at 4 °C and 5,400 ×*g* for 15 min, and the total protein concentrations in the resultant homogenate supernatant were determined using the Pierce BCA protein assay kit (Thermo Fisher Scientific K.K., Tokyo, Japan).

The samples were mixed with an equal volume of sample buffer (EzApply, ATTO Corp.), in which the total protein content was adjusted to 1 mg/mL, and denatured at 100 °C for 3 min. The samples (18 μL/well) were loaded onto 5–20% acrylamide gel (e-PAGEL HR, ATTO Corp.) at 250 V for 30 min. The proteins were then transferred to a PVDF membrane (Clear Blot Membrane-P plus, ATTO Corp.) at 153 mA for 30 min using HorizBlot (ATTO Corp.), and then blocked for 20 min at room temperature using Blocking One P (Nacalai Tesque, Inc., Kyoto, Japan). The membrane was incubated with anti-phospho-Akt (pSer473) (1 μg/mL) for 1 h and then with goat anti-rabbit IgG (0.2 μg/mL) for 1 h. The membrane was reacted with the chemiluminescent reagent (Chemi-Lumi One Super, Nacalai Tesque, Inc.), and the bands were detected using VersaDoc 5000MP (Bio-Rad Laboratories, Inc., Hercules, CA, USA). After stripping, the membrane was incubated with a pan-Akt antibody (2 μg/mL) as an internal control, for detecting the total Akt. The band intensity was quantified using ImageJ (National Institutes of Health, USA).

### Preparation of mixed solutions of exendin-4, L-penetratin, and insulin for long-term intranasal administration study

Specific amounts of exendin-4 and l-penetratin were dissolved separately in PBS (pH 6.0) with 0.001% MC as stock solutions (2.0 mg/mL and 8.0 mM, respectively). To prepare the insulin stock solution (32 IU/mL), specific amounts of recombinant human insulin were dissolved in 100 μL of 0.1 M HCl. The insulin solution was diluted in 0.8 mL of PBS (pH 6.0) with 0.001% MC, and then normalized with 100 μL of 0.1 M NaOH. Equal volumes of L-penetratin and insulin solutions were mixed gently and adjusted to 4.0 mM and 16 IU/mL, respectively. Next, the equal volumes of exendin-4 and L-penetratin/insulin solutions were mixed gently, and the final concentrations of exendin-4, L-penetratin and insulin were 1.0 mg/mL, 2.0 mM, and 8.0 IU/mL, respectively.

### Long-term intranasal administration to SAMP8 and SAMR1

Based on the characterization of cognitive dysfunction in our previous study^22^, we defined a progressive phase of dementia in SAMP8 (Fig. 1). To evaluate the therapeutic effect of nose-to-brain exendin-4 delivery on progressive memory loss, SAMP8 and SAMR1, aged 33 weeks, were administered test solutions every day according to the schemes shown in Fig. 1; in total, these animals received 30 doses.

Every morning at 10 am, mice were anesthetized by an i.p. injection of sodium pentobarbital (50 mg/kg), and restrained in a supine position. The SAMP8 were administered 5 μL of exendin-4 solution with or without L-penetratin and/or insulin, through a micropipette (Pipetman P-20, Gilson, Inc.) inserted directly into the left nostril. The doses of exendin-4, l-penetratin, and insulin were 0.125 mg/kg (1.0 mg/mL), 2.0 mM, and 1.0 IU/kg (8.0 IU/mL), respectively. The untreated control SAMP8 and SAMR1 received intranasal administration of only the vehicle (PBS pH 6.0). After each administration, mice were kept in a supine position for 15 min and then returned to their cages.

At the beginning of the study, 32 SAMP8 were divided into 4 groups (8 mice for each group) for evaluating the pharmacological effects, and 8 SAMR1 were used as a normal control. The aged SAMP8 might have been physically weakened compared with normal control SAMR1; thus, during the 1 month long-term administration study, some of the SAMP8 died, and their number were decreased. In contrast, control SAMR1 survived throughout the study, and their number remained constant.

To examine the therapeutic effects of nose-to-brain insulin delivery, the novel object recognition (NOR) and Morris water maze (MWM) tests were initiated one day after the 28^th^ and 29^th^ administrations, respectively. The NOR test was used because of its simplicity in detecting learning and memory with relatively low stress, and the MWM test was used as it is the most widely used behavioral technique used to assess the spatial learning ability of mice. As shown in Fig. 1B, a series of MWM tests were performed on the first day for pretraining and subsequently for 7 days of training sessions. During the NOR test (2 days for training and test) and MWM test period (8 days for pretraining and trainings), the mice were not administered any drugs in order to avoid exhaustion before the trials.

On the day after the NOR test (described in next section) and before the MWM test, the blood glucose concentrations of mice were monitored after the 29^th^ intranasal administration of exendin-4 with or without l-penetratin and insulin (Fig. 1B). Blood was obtained from the tail vein before and 15, 30, and 60 min after administration, and the blood glucose concentration was measured using a glucose meter (One Touch Ultra View, Johnson & Johnson K.K., Tokyo, Japan).

The day after the last trial of the MWM test, the mice were anesthetized by i.p. injection of sodium pentobarbital (50 mg/kg), and then exendin-4 with or without l- or d-penetratin insulin was administered intranasally (30^th^ administration) as described above. At 15 min after administration, blood samples were taken from the left jugular vein. Immediately after blood withdrawal, the abdominal cavity was opened, and the intravascular content in the brain was flushed by perfusion of ice-cold PBS (pH 7.4) into the left ventricle of the heart at 5.5 mL/min for 5 min using a peristaltic pump (ATTO Corp.). Right afterwards, the brain was fixed by perfusion of ice-cold 4% PFA into the brain at 5.5 mL/min for 10 min. Subsequently, the whole brain was carefully isolated and washed with ice-cold PBS (pH 7.4) and weighed after removal of the water content.

### NOR test

On 27^th^ and 28^th^ day of administrations, mice were acclimatized in the test space for NOR test, by placing them in an empty plastic container (W: 368 mm, D: 480 mm, H: 305 mm) for 10 min/day under a silent environment without any shade or objects. On the day after the 28^th^ administration, the mice were again acclimatized in the container for 10 min, and after a 2-h interval, the mice were trained in the container with two of the same plastic objects (A and B) for 5 min. After 24 h, the mice were tested in the container with an original object (A) and a novel object (C) for 5 min. During training and test periods, the time of continuous contact with both objects was recorded. The scores were expressed as a percentage of the time spent on object B or C, divided by the total time of contact with both objects (A and B/C).

### MWM test

A round gray pool (diameter: 120 cm; depth: 45 cm; Shin Factory, Fukuoka, Japan) and platform (diameter: 12.5 cm; height: 15 cm) were used for the MWM test. The pool area was divided into northeast, northwest, southeast, and southwest quadrants. The pool was placed in a silent environment with a fixed landscape by surrounding it with nontransparent screens and landmark objects, and the water was warmed to 22 ± 1 °C. The series of tests included 1 day of pretraining and 7 days of training sessions. During each session, the behavior of each mouse was tracked with a camera and analyzed using SMART version 3.0 (Panlab SLU, Barcelona, Spain).

The pretraining session was conducted a day before the first training session, to acclimatize the mice to the test conditions and to teach them how to escape to the platform. The platform was placed in the center of the pool, with the height set at 1 cm above the water surface. The mouse was placed on the platform and prompted to jump into the water. After swimming for 15 s, the mouse was guided to return to the platform. This procedure was repeated three times with rest intervals of more than 10 s for each mouse.

For the training sessions, the platform was placed in the southwest quadrant, and the height of the platform was positioned at 1 cm under the water surface, from which the mouse could escape only by using its memory. The mouse was released into the northeast, northwest, or southeast quadrant randomly, and the time taken by it to reach the platform was recorded. If the mouse could not escape to the platform within 120 s, the time was scored as 120 s, and the mouse was guided to return to the platform. To assess spatial learning, each mouse was kept on the platform for 10 s after every training trial. In the study using SAMP8 and SAMR1 with mild memory loss, the trial was repeated three times per day for 4 days. In the study using mice with progressive memory loss, the trial was repeated four times per day for 7 days.

Following the last training session, SAMP8 and SAMR1 with progressive memory loss completed the probe trial. The platform was removed from the pool, the mouse was released into the northeast quadrant, and the behavior was observed for 120 s. The number of crossings through the virtual platform was recorded.

### Measurement of exendin-4, insulin, and Aβ concentrations in plasma and brain samples

The brain samples from SAMP8 and SAMR1 with progressive memory loss were separated sagittally into the right and left sides of the brain on a silicone board. The right side was used for immunohistological staining, while the left side was homogenized with 4× volume of assay buffer (Phoenix Pharmaceuticals, Inc.), PCMB (2 mM) as the IDE, and protease inhibitor cocktail. The plasma and brain homogenates were centrifuged at 4 °C and 5,400 × *g* for 15 min, and the concentrations of exendin-4 and insulin in the resultant plasma and brain homogenate supernatants were measured using ELISA kits (exendin-4 EIA kit and Human Insulin Ultrasensitive ELISA kit, respectively. The concentrations of Aβ (1–40) and Aβ (1–42) in the plasma were measured with Mouse/Rat Aβ (1–40) and Aβ (1–42) assay kits. The absorbance at 450 nm was detected using a microplate reader (Synergy HT, BioTek Instruments, Inc., Winooski, VT, USA).

### Immunohistological staining of brain specimens

The right side of the brain obtained as described above was immersed in 4% PFA for 24 h and then moved to ice-cold PBS (pH 7.4) and stored at 4 °C. After removing the PBS, the brain was trimmed coronally to obtain 3-mm slices at the position of Bregma −1.5 mm. The brain specimen was immersed serially in the embedding cassette in 50%, 70%, 80%, and 90% ethanol for 3 h each and then immersed in 50% xylene–50% ethanol for 12 h and 100% ethanol for 3 h for delipidation. Paraffin blocks were prepared after dehydration, clearing, and subsequent paraffin infiltration.

A thin slice at Bregma −2.0 mm was obtained and mounted on a glass slide, deparaffinized, and immersed in citrate buffer for 40 min in a water bath at 98 °C to activate the antigen. The slice was washed in citrate buffer, treated with 3% hydrogen peroxide for 10 min at room temperature, washed, and immersed in blocking reagent for 10 min at room temperature to inhibit nonspecific reactions. The slice was incubated with primary antibody (anti-NeuN antibody or anti-Aβ (1–42) antibody) diluted appropriately for staining overnight at 4 °C. After washing, the slice was treated with a polymer reagent (ENVISION + , anti-rabbit antibody, Dako Denmark A/S, Glostrup, Denmark) for 30 min at room temperature and then exposed to 3,3′-diaminobenzidine (DAB) to produce the chromogenic reaction. The stained brain tissue was observed by microscopy (Olympus BX51 and DP71), and the NeuN-positive cell numbers in the hippocampus were analyzed using WinROOF (Mitani Corp., Tokyo, Japan). Throughout the immunohistological examinations, brain specimens were blinded to avoid experimenter bias in counting cells.

### Statistical analysis

Each value is expressed as the mean and standard error of the mean (SEM) of multiple determinations. The significance of differences in the mean values of two groups was evaluated using Student’s unpaired *t*-test. For multiple comparisons with the control group, analysis of variance (ANOVA) with Dunnett’s test was used. IBM SPSS Statistics Version 22 (IBM Corp., Armonk, NY, USA) was used for statistical analysis. Differences were considered significant at *p* value < 0.05.

## Electronic supplementary material

Supplementary information
