# Supplementary material for: Effective nose-to-brain delivery of exendin-4 via coadministration with cell-penetrating peptides for improving progressive cognitive dysfunction
Source: Sci Rep. 2018 Dec 5;8:17641. doi: 10.1038/s41598-018-36210-9 (PMC6281676; doi:10.1038/s41598-018-36210-9)
Supplement: Supplementary file 1 — Supplementary information [file 41598_2018_36210_MOESM1_ESM.pdf]

Supplementary Information:

SCIENTIFIC REPORTS

Effective nose-to-brain delivery of exendin-4 via coadministration with cell-penetrating peptides for improving progressive cognitive dysfunction

Noriyasu Kamei, Nobuyuki Okada, Takamasa Ikeda, Hayoung Choi, Yui Fujiwara, Haruka Okumura, Mariko Takeda-Morishita*

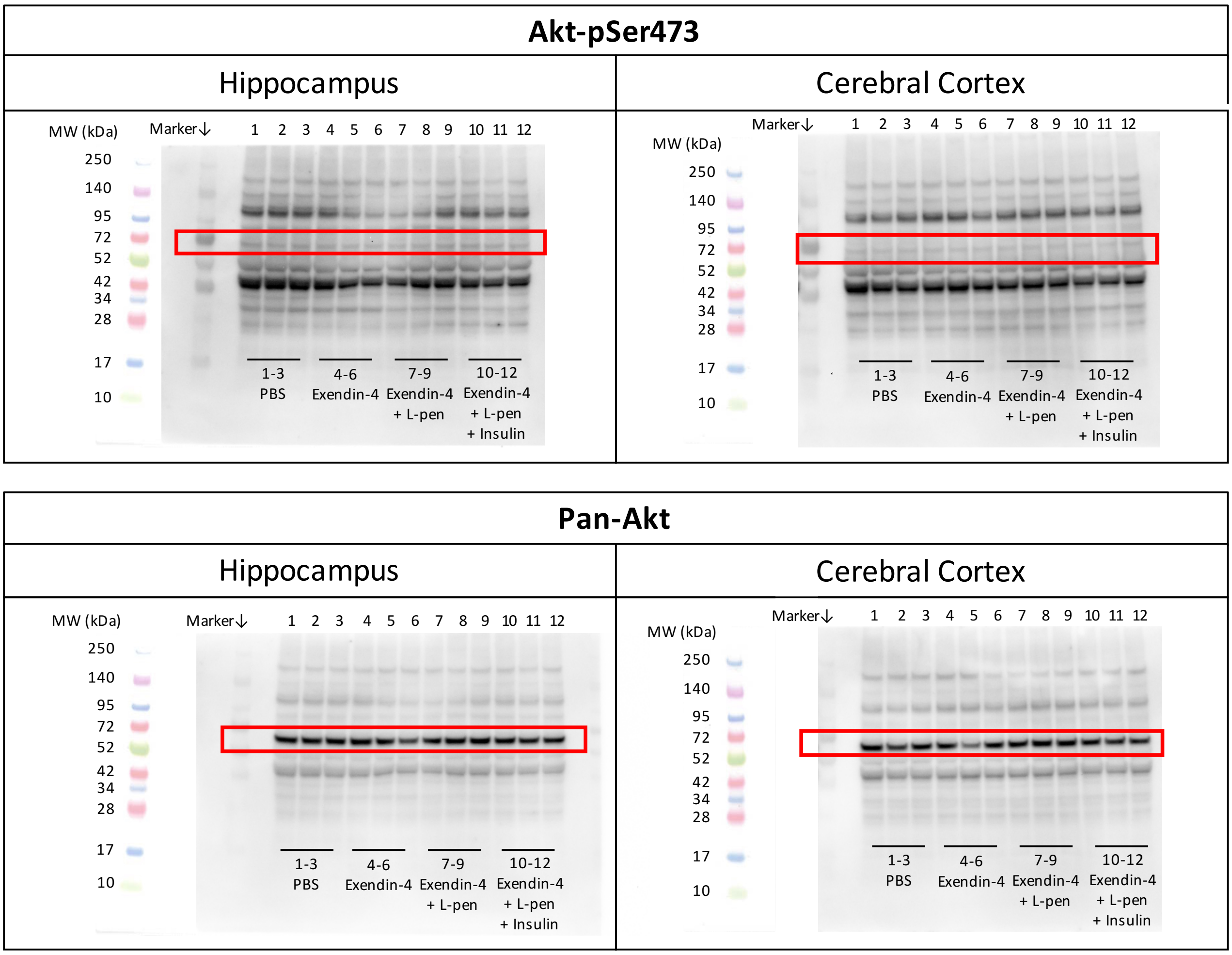

Figure S1. Original full-length data of chemiluminescent acquisition of the immuno-blotted membrane with anti-Akt-pSer473 or anti-pan-Akt antibodies. Each sample for loading to gel electrophoresis were obtained by extraction from the homogenate of the hippocampus and cerebral cortex after single intranasal administration of exendin-4 with L-penetratin and insulin to male ddY mice.
